# Tradeoffs and Compatibilities of Chemical Properties in C_p_H_q_F_r_O_s_ System

**DOI:** 10.1038/s41598-019-46562-5

**Published:** 2019-07-19

**Authors:** Yasuharu Okamoto

**Affiliations:** Materials Data Platform Center, Research and Services Division of Materials Data and Integrated System, National Institute for Materials Science, 1-2-1 Sengen, Tsukuba, Ibaraki 305-0047 Japan

**Keywords:** Cheminformatics, Computational chemistry

## Abstract

To comply with the Kigali amendment to the Montreal Protocol in 2016, development of new refrigerants with low global warming potential is urgently required in addition to satisfying the conventional requirements of cooling performance, safety, and non-destructiveness to the ozone layer. Because these requirements closely correlated, the proper control of various chemical properties is necessary to fulfill the requirements. However, simultaneous satisfaction of all the requirements is extremely difficult because of the tradeoffs among the chemical properties. Hence, we must correctly recognize how chemical properties behave when the composition of molecules is changed. We performed an in-silico screening that combines quantum chemical calculations, machine learning, and database search, where 10,163 molecules were investigated exhaustively within the properly imposed constraints; subsequently we found a few candidates.

## Introduction

The elucidation of tradeoffs among various design factors is essential in material design. A tradeoff may be included in a single design factor. For example, the behavior of the turnover rate of a catalytic reaction is often characterized by a volcano diagram that is formed by the competition between the ease of adsorption of reactant molecules on the catalyst surface and the facile desorption of product molecules from the surface to enable successive reactions^1^. However, the tradeoffs among a large number of design factors are more important and inevitable in material design. The optimal combination of the design factors is known as the Pareto front. Mathematically, considering the Pareto front is enough to obtain the optimal solutions; however, it is difficult to obtain the Pareto front because the relationship between the design factors in the possible compositions of the target material is highly complicated; this is because the factors demonstrate a dependent relationship in some cases while they are sometimes competing in another cases. Moreover, it is difficult to experimentally investigate the possible compositions exhaustively.

It is noteworthy that the importance of in silico screening for obtaining the optimal compositions of molecules/crystals in material design is increasing with the recent development of computers and computing technologies^2–4^. Refrigerants are suitable for such in silico screening because the molecule size is relatively small: they must be a gas at room temperature and atmospheric pressure. This enables us to examine the chemical space of the refrigerants by exhaustively changing their compositions within the range of the appropriately determined constraints. Such an exhaustive search helps to reduce type-II errors or false negatives, and may yield an unexpected discovery.

The specifications required for refrigerants have increased with the times. Chlorofluorocarbons (CFCs) were widely used as ideal refrigerants because its cooling performance is compatible with the safety requirements. However, because the Cl radicals produced through the decomposition of CFCs by ultraviolet light in the upper atmosphere destroyed the ozone layer in a chain reaction manner, the use of CFCs and hydrochlorofluorocarbons (HCFCs) was phased out by the Montreal Protocol in 1987^5^. Hence, the primary targets for new refrigerants have shifted from CFCs and HCFCs to hydrofluorocarbons (HFCs). However, many HFCs are stable in the atmosphere; therefore, they possess an extremely high global warming potential (GWP), as much as ten thousand times GWP of CO_2_ in worse cases. The GWP of CO_2_ is defined as 1. The phasedown of the use of HFCs was decided in the Kigali Amendment to the Montreal Protocol in 2016^5^. Therefore, while maintaining the necessary performance and safety requirements, a new refrigerant that does not destroy the ozone layer with a low GWP is urgently required.

In this study, the in-silico screening of new refrigerants was performed by combining quantum chemical calculation, machine learning, and database searching. Here, the heat of vaporization (ΔH_vap_) was chosen as a performance indicator. Refrigerants with higher ΔH_vap_ yield efficient cooling/heating processes. The safety of a refrigerant was tested by calculating the heat of combustion (H_c_). A smaller exothermicity is better as a refrigerant. The ozone non-destructive property was treated by creating the refrigerant from C, H, F, and O elements without including Cl and Br as the constituents. As for the GWP, if the refrigerant has at least one C=C or C≡C bond, its atmospheric lifetime becomes short and does not affect global warming. Meanwhile, when an unsaturated bond does not exist, the GWP must be predicted by machine-learning approaches to select molecules that have a low GWP.

## Methods

### Calculation of heat of combustion

In the combustion process of C_p_H_q_F_r_O_s_, the most important chemical sink of fluorine is HF; subsequently, the excess H atoms are oxidized to H_2_O. Conversely, when the number of H is not enough to form HF, the remaining F becomes COF_2_. COF_2_ formation is prioritized over CO_2_ formation. Therefore, the chemical equations of C_p_H_q_F_r_O_s_ combustion are expressed as follows^6^:

1 $$\begin{array}{ll}{C}_{p}{H}_{q}{F}_{r}{O}_{s}+\frac{(4p+q-r-2s)}{4}{O}_{2}\to pC{O}_{2}+\frac{(q-r)}{2}{H}_{2}O+rHF & (q\ge r)\end{array}$$

2 $${C}_{p}{H}_{q}{F}_{r}{O}_{s}+\frac{(4p+q-r-2s)}{4}{O}_{2}\to \frac{(2p-r+q)}{2}C{O}_{2}+\frac{(r-q)}{2}CO{F}_{2}+qHF\,(q < r)$$

The enthalpy change at 298.15 K of these reactions was calculated at the B3LYP/6–31 G(d, p) level of theory. All quantum chemical calculations were performed using the Gaussian 16 suite of programs^7^. Following the convention of refrigerant research, exothermic reactions were designated as positive.

### Estimation of heat of vaporization and boiling point

The heat of vaporization (ΔH_vap_) can be calculated from the free energy difference of molecules between the liquid and gaseous states in the framework of quantum chemistry. However, this approach requires a dielectric constant to calculate the solvent effects based on the polarized continuum model or other self-consistent reaction field methods^8^ .Such an approach cannot be applied to the present screening study because we must treat many virtual molecules whose dielectric constant is unknown. Instead, we used the Joback method (JBM) to estimate the heat of vaporization^9^. The JBM is a type of group contribution method where molecular properties are expressed by the sum of contributions determined by dividing a molecule into its partial structures. Although the JBM often fails to distinguish isomers and may not be suitable to distinguish the subtle differences of chemical structures, it is convenient and advantageous in predicting the boiling point (BP) using the same groups.

### Estimation of GWP

As stated above, the prediction of the GWP is necessary when no unsaturated bond exists between the carbon atoms. We used the GWP of over 100 years as the GWP in this study. The fifth assessment report (AR5) by an intergovernmental panel on climate change (IPCC) lists lifetimes, radiative efficiencies, and metric values of more than 200 green house molecules in Appendix 8.A^10^. Among them, 131 molecules that consist of C, H, F, and O elements and that do not have a C=C double bond were selected to construct a regression model using gradient boosting regression (GBR). GBR in this study was based on the scikit-learn package, which is a collection of application program interfaces for machine learning in Python^11^. The details of GBR are as follows: 131 pieces of data were divided into 4:1 for training set and test set. The same partial molecular structures as the JBM were used as the descriptors for GBR except for the -COOH and -COO- groups. The -COOH and –COO– groups were calculated as >C=O plus –OH, and >C=O plus –O–, respectively, because no molecule in the AR5 data contains -COOH or -COO- groups. Meanwhile, –COOH and –COO– groups were used in the estimation of ΔH_vap_ and BP by the JBM. We used the least-squares regression as the loss function (loss = “ls”). The learning rate, at which the contribution of each decision tree shrinks, and the number of boosting stages to perform were set conservatively to avoid overfitting (learning rate = 0.0001 and n_estimators = 100000). In addition, the maximum depth, minimum samples split, and maximum feature parameters of the scikit-learn gradient-boosting regression module (ensemble.GradientBoostingRegressor) were set as 6, 3, and 2, respectively.

## Results and Discussion

**Figure 1:**
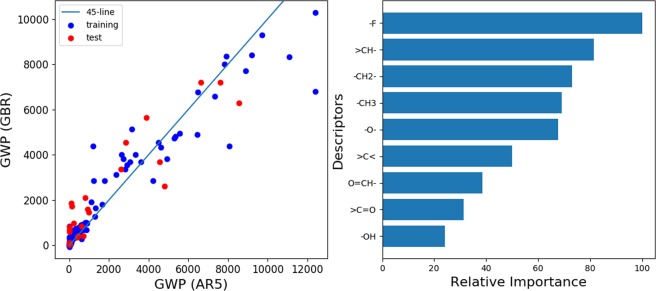
(Left panel) Scatter chart of predicted GWP by gradient-boosting regression (GBR) method versus GWP tabulated in fifth assessment report (AR5) by intergovernmental panel on climate change. (Right panel) Relative importance of partial structure groups in GWP prediction by GBR.

Figure 1 (left panel) shows a scatter chart of the predicted GWP by GBR versus GWP in AR5. The 45° line means a perfect fitting. We also show the following data in Spreadsheet S1, which is attached as supplementary datasets: Chemical formula of 131 molecules, descriptors for GBR, GWP tabulated in AR5 by IPCC, and the predicted GWP. It appears that the regression model predicts the GWP in AR5 well. The R^2^ scores for the training and test sets were 0.899 and 0.836, respectively. Figure 1 (right panel) shows the relative importance of the partial structure groups used as descriptors in GBR. It is noteworthy that the contribution of −F is the greatest for the GWP. Meanwhile, O=CH–, >C=O, and the –OH groups are not significant in the GWP. These groups appear to be unstable in the upper atmosphere. As the number of hydrogen atoms bonding to a carbon atom increases from >CH– to –CH_3_, the importance of the GWP decreases. These results suggest that as the number of hydrogen atoms and/or specific functional groups (O=CH–, >C=O, and –OH) increases, the atmospheric lifetime shortens, which in turn decreases the GWP.

Next, we discuss the upper limit of the molecular weight (M_w_) of the target refrigerants in this study. In practice, the upper limit of M_w_ is inevitably compromised by the balance of available computing resources and computational cost. Effectively, the CPU time of the *ab-initio* calculation is proportional to the cube of the total number of electrons of the molecule. Therefore, the computational cost increases rapidly as the molecular size increases.

We imposed the following constraints for the investigation of new refrigerants

- The molecule contains at least one fluorine atom to yield better flame retardancy.
- The molecule does not have one or more successive O–O bond to maintain chemical stability.
- At the most, one OH group is bonded to one carbon atom.
- The molecule does not have ring structures and/or carbon side chains.
- Geometric isomers such as cis and trans forms and optical isomers are not distinguished.

By classifying the number of π bonds between the carbon atoms (N[π]), the relationship between the upper limit of M_w_ and the number of possible molecular formulas is shown in Table 1. As the molecular weight increases, the number of possible molecular formulas increases exponentially, which is expressed as 1.14 × exp (0.05 × M_w_). In addition, as the molecular size increases, the number of possible isomers increases within the same molecular formula. In the case of M_w_ ≤ 125, which was adopted in this study, we observed that 10,163 molecules were generated from 647 molecular formulas. Although we know that there are refrigerants of M_w_ > 125 such as CF_3_CF_2_OCH_3_ (HFE-245mc: M_w_ = 150.05), exhaustive calculations of possible compositions become difficult as the number of F atoms in the molecule increases. To include molecules with a larger molecular weight, it is necessary to predict the enthalpy of molecules based on machine-learning approaches such as high-dimensional neural network potentials^12^ instead of directly calculating the enthalpy by *ab-initio* calculations.

**Table 1:** Relationship between upper limit of molecular weight and the number of possible molecular formulas with respect to the number of π bonds in the molecule N[π].

		upper limit of molecular weight						
		50	75	100	112	125	137	150
N[π]*	0	4	15	62	102	153	222	338
	1	1	7	33	62	102	153	241
	2	1	12	43	79	142	229	364
	3	0	5	25	43	79	142	242
	4	0	2	25	43	72	132	240
	5	0	0	3	25	43	72	141
	6	0	0	3	15	37	62	120
	7	0	0	0	3	15	37	79
	8	0	0	0	0	4	20	52
	9	0	0	0	0	0	4	20
	10	0	0	0	0	0	0	5
total		6	41	194	372	647	1073	1842

^*^Number of π bonds between carbon atoms. C=C double bond was counted as one π bond whereas a C≡C triple bond was counted as two π bonds.

We consider it desirable to generate data by exhaustive calculations with as few constraints as possible within the determined range of the molecular size to reduce the risk of type-II errors or false negatives. This may include somewhat irrelevant molecules as flame retardant refrigerants whose ratio of (number of H atoms)/(number of F atoms) is much greater than one; however, they can be removed by using the appropriate filters. In addition, this work is envisaged as a type of materials informatics that combines materials science and data science, where the amount of data is important for future studies involving fields other than refrigerants.

We also considered the upper limit of M_w_ from a completely different perspective. According to Trouton’s rule^13^, the heat of vaporization (ΔH_vap_) must be 25 kJ/mol or less if the molecule exists as gas at room temperature. In addition, ΔH_vap_ must be beyond 200 kJ/kg to satisfy the performance requirement for new refrigerants. The values of ΔH_vap_ of widely used refrigerants are 164, 208, 217, 275, and 382 kJ/kg, for HFC-125, R404A, R134a, R410A, and R32, respectively. These considerations lead to the inequality of M_w_ as follows:

3 $$25[\frac{kJ}{mol}]\times \frac{1000}{{M}_{W}} > 200[\frac{kJ}{kg}]\Rightarrow {M}_{W} < 125$$

The heat of combustion (H_c_) was obtained by calculating the enthalpy change at the standard state with respect to 10,163 molecules. In addition, ΔH_vap_ was estimated using the JBM. To ensure the traceability of this study, we listed the following data of all molecules in Spreadsheet S2, which is attached as supplementary datasets: condensed formula, molecular formula, molecular weight, N[π], enthalpy, and Gibbs energy at the standard state, H_c_, ΔH_vap_ by the JBM, BP by the JBM, GWP predicted by GBR using JBM groups as descriptors (N[π] = 0 only), and the assignment of JBM groups.

**Figure 2:**
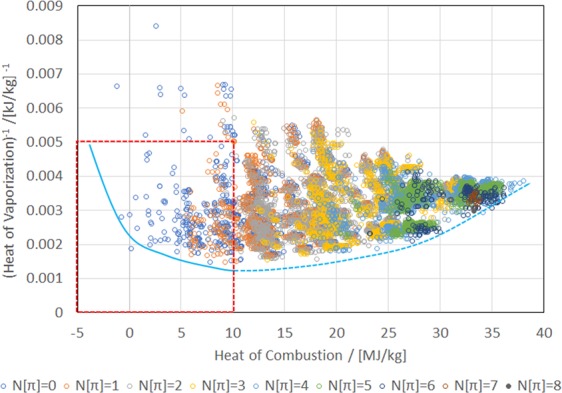
Scatter chart of (heat of vaporization)^−1^ (ΔH_vap_)^−1^ versus heat of combustion of 10,163 molecules distinguished by the number of π bonds N[π]. Dotted red lines show the area desirable for refrigerants. See text for the explanation of solid and dotted blue lines.

Figure 2 shows a scatter chart of (ΔH_vap_)^−1^ versus H_c_, where the inverse of ΔH_vap_ is plotted because multi-objective optimization is typically formulated as a minimization problem. As the number of π bonds in a molecule increases, the heat of combustion tends to increase, but it does not increase monotonically at N[π] ≥ 4. This is because although the molecule is destabilized as the number of its π bonds increases, the increase in the unsaturated bonds means a decrease in the number of hydrogen atoms in the molecule. It is noteworthy that if the same weight is given, the heat of combustion of hydrogen is greater than that of carbon. In the evaluation of ΔH_vap_ by the JBM, the presence of functional groups such as –OH, –COOH, –COO, O=CH– significantly increases the heat of vaporization. In particular, the OH group is advantageous for obtaining a high ΔH_vap_ in terms of per unit weight. In fact, as will be discussed below, most of the molecules that constitute the lower limit of (ΔH_vap_)^−1^ contain one or more OH groups. Conversely, F is the only functional group whose contribution to ΔH_vap_ becomes negative in JBM parameterization. Thus, a molecule that consists of many F atoms results in a smaller ΔH_vap_. For example, the molecule that yields the maximum value of (ΔH_vap_)^−1^ (0.0084 in the figure) is CHF_2_CF_3_. Although molecules that consist of many π bonds must have many carbon atoms, the number of F atoms in such molecules will be small owing to the constraint of the molecular weight (M_w_ ≤ 125). Therefore, the upper limit of (ΔH_vap_)^−1^ decreases as the number of π bonds increases. The solid blue line in the figure represents a tradeoff between G_c_ and (ΔH_vap_)^−1^, that is, when the performance of one is improved, the performance of the other worsens. Meanwhile, the dotted blue line means a region where H_c_ and (ΔH_vap_)^−1^ can be improved (or worsened) simultaneously. What is desirable as a refrigerant is a molecule in an area of dotted red lines that simultaneously satisfies both low heat of combustion and high heat of vaporization.

The flammability of refrigerants is classified into four categories in ISO 817: Class 1 (no flame propagation), Class 2L (lower flammability), Class 2 (flammable), and Class 3 (higher flammability)^6^. Practical refrigerants belong to Class 1 or Class 2L. These categories are determined based on the exhibition of flame propagation, lower flammability limit (LFL), heat of combustion, and burning velocity. Therefore, it may be inadequate to discuss inflammability based on combustion heat only; nonetheless, the combustion heat is easily obtained from *ab-initio* calculations and can be an appropriate indicator of screening for flammability. If a refrigerant has a heat of combustion that is 19 MJ/kg or larger, it belongs to Class 3. Meanwhile, a refrigerant whose heat of combustion is less than 19 MJ/kg falls into Classes 1–3 depending on flame propagation, LFL, and burning velocity. The threshold of 19 MJ/kg appears to be high. Thus, we set the tolerance of H_c_ to 10 MJ/kg. This is based on the observation that the heats of combustion of R32 (CH_2_F_2_), R143a (CH_3_CF_3_), R1234yf (CF_3_CF=CH_2_), and R1234ze(E) (CF_3_CH=CHF) that are representative refrigerants belonging to class 2L are 9.5, 10.2, 10.7 and 10.1 MJ/kg, respectively^14^. R32 is the latest refrigerant primarily used for room air-conditioners; nevertheless, its GWP is as high as 677^10^. Therefore, a new refrigerant is eagerly anticipated to reduce the impact on global warming.

The molecules satisfying N[π] ≥ 1 have at least one C=C or C≡C bond that renders their atmospheric lifetime short enough such that their impact on global warming is negligible. However, only a small number of molecules have passed the tests of H_c_ (≤10 MJ/kg) and ΔH_vap_ (≥200 kJ/kg). The numbers of molecules that passed the tests were 28 and 2 for N[π] = 1 and 2, respectively. We found that no molecule passed the tests when N[π] is three or higher. It is noteworthy that we excluded the molecules containing the enol (>C=C(OH)–) group and similar groups in the triple bond (–C≡COH). As stated above, although the molecules containing the OH group have the advantage of increasing ΔH_vap_, the enol group is chemically unstable and is not a realistic proposal for a new refrigerant. In addition, we excluded the molecules containing the carboxy (–COOH) group. Molecules containing the –COOH group exhibit an extremely high BP. Even the lightest formic acid boils above 100 °C.

Regarding the molecules of N[π] = 0, the GWP must be tested in addition to testing the H_c_ and ΔH_vap_. Because of the limited number of available GWP data in AR5 by the IPCC, and the difficulty in constructing a good regression model at a low GWP, we imposed a rather loose criterion with an allowance of 1000 or less with respect to the predicted GWP to reduce the risk of type-II errors. The number of molecules with N[π] = 0 that passed the tests of GWP, H_c_, and ΔH_vap_. is 141. It is noteworthy that we excluded the molecules containing the carboxy (–COOH) group. The list of molecules that passed the H_c_ and ΔH_vap_ tests are shown in Spreadsheet S3, which is attached as supplementary datasets where the condensed formula, N[π], H_c_, ΔH_vap_, and BP predicted by the JBM are shown. We consider a BP lower than 0 °C to be desirable in practical refrigerants. In addition, the GWP predicted by GBR is shown for molecules that satisfy N[π] = 0. We also listed the following results of search by SciFinder (if any): CAS registry number, CAS name and experimental/predicted BP.

Table 2 summarizes the results of a series of screening tests. The first eight molecules in the table satisfied all four criteria of H_c_ (≤10 MJ/kg)_,_ ΔH_vap_ (≥200 kJ/kg), GWP (≤1000), and BP (≤0 °C). However, the experimental BP of CH_2_FOCH_2_F is 32–34 °C. Therefore, the molecule is not suitable as a refrigerant. It is noteworthy that there are four known refrigerants in them (CH_2_F_2_ [R32], CHF_2_CH_2_F [R143b], CHF_2_OCH_3_ [HFE-152a], and CF_2_=CHF [R1123]). Further, using R1123 alone is not stable under specific conditions owing to the disproportionate reaction of CF_2_ = CHF → 0.5 CF_4_ + 1.5 C + HF^15^. In addition, difluoroketene (CF_2_=C=O) was reported as a reactive and unstable compound^16^. In general, because ketenes are reactive, O=C=CFCHF_2_ and O=C=CHCF_3_ require further examination in terms of stability.

**Table 2:** Molecules that passed screening tests with respect to heat of combustion (H_c_), heat of vaporization (ΔH_vap_), boiling point (BP), and global warming potential (GWP), and three additional molecules discussed in text (CF_3_OCHO, CHF_2_OCH_2_OH, and CF_3_OCH_2_OH). If any, CAS registry number, and experimental/predicted BP by SciFinder are also shown.

Condensed formula	H_c_* [MJ/kg]	ΔH_vap_ [kJ/kg] (Joback)	BP [°C] (Joback)	GWP**	CAS reg. no.	BP [°C] (SciFinder)
CH_2_F_2_	7.23	311.1	−52.3	822	75-10-5	−51.6***
CHF_2_CH_2_F	8.90	204.7	−30.6	744	430-66-0	5***
CHF_2_OCH_3_	9.08	249.0	−7.5	226	359-15-9	−4***
CH_2_FOCH_2_F	9.88	253.8	−7.0	231	462-51-1	32–34***
CF_2_=CHF	8.30	215.0	−26.1	n.a.	359-11-5	−78***
CF_2_=C=O	8.37	316.2	−35.4	n.a.	683-54-5	−69.7 ± 35.0
O=C=CFCHF_2_	8.84	233.5	−13.7	n.a.	n.a.	n.a.
O=C=CHCF_3_	7.71	224.5	−16.4	n.a.	134736-46-2	24.1 ± 40.0
CF_3_OCHO	1.74	223.0	37.7	893	85358-65-2	−2.0 ± 40.0
CHF_2_OCH_2_OH	6.02	378.5	84.7	124	188487-13-0	−21.6 ± 30.0
CF_3_OCH_2_OH	3.46	305.0	81.2	83	134736-46-2	−51.8 ± 35.0

^*^Calculation at B3LYP/6–31G(d,p) level of theory.

^**^Predicted value by gradient-boosting regression method for N[π] = 0 molecules.

^***^Experimental BP listed in SciFinder.

We observed that the predicted BPs by the JBM are sometimes vastly different from those listed in the SciFinder database. For example, according to SciFinder, the BPs of CF_3_OCHO, CHF_2_OCH_2_OH, and CF_3_OCH_2_OH molecules are lower than 0 °C and they satisfy the four criteria above. These predicted BPs by SciFinder exhibit error bars of ±30 °C or more. Nonetheless, the BP of CF_3_OCH_2_OH predicted by SciFinder is low and CF_3_OCH_2_OH will remain as gas at 0 °C even if the error bar is considered.

Although we have discovered a few candidates for new refrigerants, they include molecules that might exhibit stability problems like ketene. Therefore, further research is required to increase the number of good candidates. According to Spreadsheet S2 (supplementary datasets), more than 90% of 10,163 molecules examined in this study satisfy the respective criterion of ΔH_vap_ and GWP. However, only 3.7% and 0.8% of molecules satisfy the criteria of H_c_ and BP, respectively. When the threshold of H_c_ is relaxed to 12.5 MJ/kg, molecules of 10.2% pass the criterion; however, even if the threshold of BP is increased to 10 °C, the number of molecules satisfying the criterion remains at 1.2%. Because the estimated value of the BP has large uncertainties as described above, it appears that an accurate BP estimation method becomes essential for efficiently selecting the candidate molecules.

**Figure 3:**
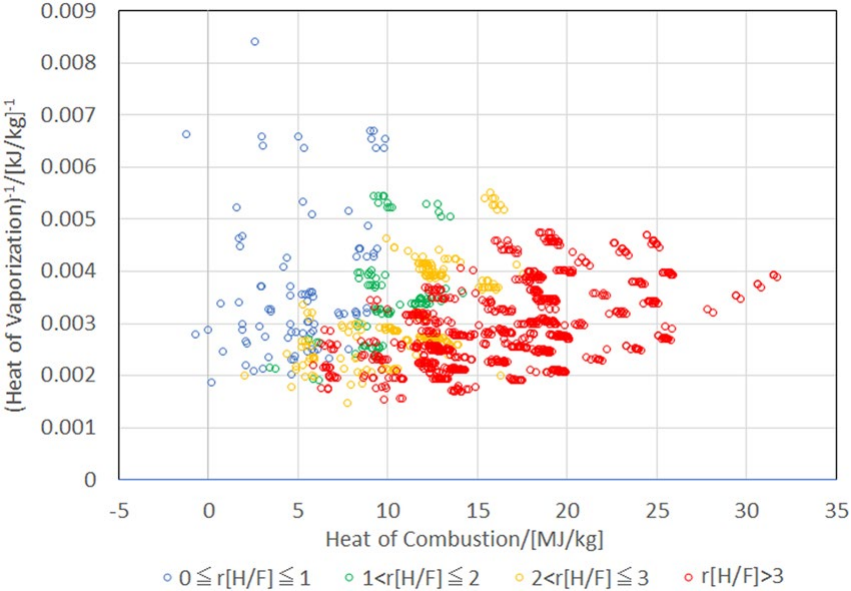
Scatter plot of (heat of vaporization)^−1^ (ΔH_vap_)^−1^ versus heat of combustion (H_c_) of 1357 molecules with N[π] = 0 distinguished by the ratio of number of H atoms to number of F atoms (r[H/F]) in a molecule.

Finally, we discuss the tradeoffs of chemical properties in the C_p_H_q_F_r_O_s_ system. As shown in Fig. 3, the presence of fluorine lowers the heat of combustion, which is advantageous for refrigerants; however, as shown in Fig. 1, it tends to exacerbate the GWP owing to the strong C-F bonds. This is a well-known tradeoff in the refrigerants field. Moreover, according to the parameterization by the JBM, fluorine contributes to lowering the heat of vaporization. The effect of F on the BP cannot be decided unconditionally because cases of both BP increase and decrease exist by increasing the number of F in the molecule. Although the presence of π bonds between carbon atoms increases the heat of combustion, it reduces the atmospheric lifetime significantly; therefore, the GWP is not a problem. The influence of the π bonds on the heat of vaporization as well as the BP appear to be neutral. Specific functional groups such as –COOH, –COO–, >C=O, and -OH decrease the heat of combustion because they are already partly oxidized. They increase the heat of vaporization significantly, which is especially true of the –OH group. These functional groups are expected to be vulnerable to the attack by radicals and/or ultraviolet light in the upper atmospheric environment, which decreases the GWP. In fact, O=CH–, >C=O, and the –OH groups are not significant in the GWP predicted by GBR (Fig. 1). However, they increase the BP. It appears unlikely that molecules containing –COOH or –COO– groups remain in the gas state at room temperature. These results are summarized in Table 3.

**Table 3:** Effect of partial chemical structures (F,πbonds, and specific functional groups) on heat of combustion (H_c_), heat of vaporization (ΔH_vap_), global warming potential (GWP), and boiling point (BP).

	Hc	ΔH_vap_	GWP	BP
F	Decrease (f*)	Decrease (u*)	Increase (u)	Neutral
π bonds (C=C/C≡C)	Increase (u)	Neutral	Decrease (f)	Neutral
Specific functional groups (COOH, COO, CO, OH)	Decrease (f)	Increase (f)	Decrease (f)	Increase (u)

*f/u in parenthesis means favorable/unfavorable change for design of refrigerants.

**Figure 4:**
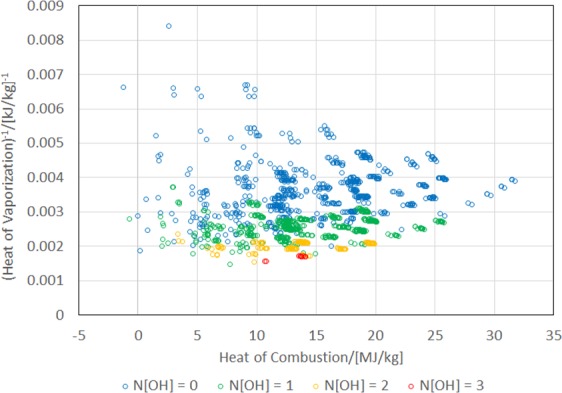
Scatter plot of (heat of vaporization)^−1^ (ΔH_vap_)^−1^ versus heat of combustion (H_c_) of 1357 molecules that satisfy N[π] = 0 distinguished by number OH groups in molecule N[OH] = 0, 1, 2, and 3.

A refrigerant not containing Cl is desirable in terms of it non-destroying nature to the ozone layer; however, it is difficult to achieve both high heat of vaporization and low BP without worsening the GWP. In the JBM, –F and –Cl groups contribute to ΔH_vap_ by −0.67 and +4.532 kJ/mol, respectively. In addition, the contributions of –F and –Cl groups to the BP is −0.03 and +38.13 K, respectively. Therefore, the heat of vaporization might be increased without significantly increasing the BP by the introduction of a Cl atom. The functional group that can replace Cl is OH. However, if OH coexists with a C=C double bond, it tends to result in a non-chemical structure such as the enol group; therefore, it will be efficient to scrutinize molecules that do not contain C=C or C≡C bonds. Figure 4 shows the (H_c_, (ΔH_vap_)^−1^) plot that is distinguished by the number of –OH groups in the molecules that satisfy N[π] = 0. Clearly, ΔH_vap_ increases with the number of OH. However, a molecule can have, at the most, one OH group to remain in a gaseous state at room temperature because of hydrogen bonds arising from the OH group. As a second-best measure, it may be possible to shorten the atmospheric lifetime of Cl containing molecules significantly by introducing a C=C double bond. This approach might satisfy the need of a low GWP without destroying the ozone layer.

In summary, by combining quantum chemical calculation, machine learning, and database search, we performed a series of computational screening for new refrigerants that satisfied four criteria: H_c_ ≤ 10 MJ/kg, ΔH_vap_ ≥ 200 kJ/kg, GWP ≤ 1000, and BP ≤ 0 °C. We examined 10,163 molecules that consisted of C, H, F, and O whose molecular weight was 125 or less. In addition to some of the current popular refrigerants, we found a few candidate molecules although further examination is required to study their stability and uncertainty in BP. To improve the screening performance, it is essential to describe the tradeoffs more accurately between these criteria. This is particularly true of the BP estimation. Moreover, it may be necessary to predict H_c_ by a machine-learning approach to reduce the cost of *ab-initio* calculations to examine molecules of larger molecular weights.
